# Plasma and Urinary Platelet Factor 4 as Biomarkers for Cardiovascular Risk in Children with Chronic Kidney Disease

**DOI:** 10.3390/biomedicines11123318

**Published:** 2023-12-15

**Authors:** Chien-Ning Hsu, Wei-Ting Liao, Wei-Ling Chen, Guo-Ping Chang-Chien, Sufan Lin, You-Lin Tain

**Affiliations:** 1 Department of Pharmacy, Kaohsiung Chang Gung Memorial Hospital, Kaohsiung 833, Taiwan; cnhsu@cgmh.org.tw; 2 School of Pharmacy, Kaohsiung Medical University, Kaohsiung 807, Taiwan; 3 Division of Pediatric Nephrology, Kaohsiung Chang Gung Memorial Hospital, Kaohsiung 833, Taiwan; winona0409@cgmh.org.tw (W.-T.L.); weilingchen@cgmh.org.tw (W.-L.C.); 4 Institute of Environmental Toxin and Emerging-Contaminant, Cheng Shiu University, Kaohsiung 833, Taiwan; guoping@csu.edu.tw (G.-P.C.-C.); linsufan2003@csu.edu.tw (S.L.); 5 Center for Environmental Toxin and Emerging-Contaminant Research, Cheng Shiu University, Kaohsiung 833, Taiwan; 6 Super Micro Mass Research and Technology Center, Cheng Shiu University, Kaohsiung 833, Taiwan; 7 College of Medicine, Chang Gung University, Taoyuan 330, Taiwan; 8 Institute for Translational Research in Biomedicine, Kaohsiung Chang Gung Memorial Hospital, Kaohsiung 833, Taiwan

**Keywords:** chronic kidney disease, cardiovascular disease, plasma factor 4, ambulatory blood pressure monitoring, children, nitric oxide, hypertension

## Abstract

Children suffering from chronic kidney disease (CKD) have a high risk of cardiovascular disease (CVD). The early detection and diagnosis of subclinical CVD in pediatric CKD can reduce mortality later in life. Plasma factor 4 (PF4) is a chemokine released by activated platelets. We examined whether or not PF4 in the plasma and urine, its kidney function normalized ratio, and fractional excretion have differential associations with CVD risk markers in 139 youths aged 3 to 18 years old with CKD stages G1–G4. Significant negative correlations were observed between plasma PF4 and cardiovascular surrogate markers, such as the left ventricular mass index (LVMI), carotid intima–media thickness (cIMT), and pulse wave velocity (PWV). The plasma PF4/creatinine (Cr) ratio was lower in CKD children with a high daytime BP and 24 h BP, high BP load, and nocturnal non-dipping status. After adjusting for confounders, the plasma PF4 and plasma PF4/Cr ratio still independently predicted an abnormal ABPM profile. In addition, both the plasma PF4 and plasma PF4/Cr ratio presented a negative correlation with the L-arginine and asymmetric dimethylarginine ratio. These findings provide convincing evidence supporting the link between PF4 and CVD markers in pediatric CKD. Our study highlights the importance of further research to assess the performance of PF4-related biomarkers in predicting CVD events and CKD progression in children with CKD.

## 1. Introduction

Cardiovascular disease (CVD) is the leading cause of death in children with chronic kidney disease (CKD) [1,2]. Even though overt CVD scarcely occurs in children, atherosclerotic vascular changes can start as early as childhood. CKD is complex in both adults and children, but a major difference between both populations is the etiology. Unlike adult CKD, the top cause of pediatric CKD is congenital anomalies of the kidney and urinary tract (CAKUT), accounting for approximately 50% [3]. Although many risk factors have been used to identify the individual risk for CVD in adult CKD [4], these may not be applied in pediatric populations because of the different etiologies [5].

Recently discovered new biomarkers [6] and several noninvasive procedures, such as 24 h ambulatory blood pressure monitoring (ABPM) [7], carotid intima–media thickness (cIMT) [7], left ventricular mass index (LVMI) [8], and pulse wave velocity (PWV) [9], are accessible for estimating the risk for CVD in pediatric patients with CKD. Using a proteomic approach, our prior work screened proteins expressed differentially between CKD children with and without an abnormal ABPM profile and identified several candidate proteins [10]. One of them is protein factor 4 (PF4).

PF4, also called CLCX4, is an 8 kDa chemokine produced by megakaryocytes and stored within platelets [11]. PF4 can neutralize heparin-induced anticoagulation, promote leukocyte adhesion to the endothelium, increase endothelial permeability, and promote inflammation and fibrosis [11,12,13]. Atherosclerotic plaque formation involves the transport of PF4 from the blood into deeper layers of the vessel [14]. In CKD, either an increase or no change in the plasma concentration of PF4 has been reported in adult patients receiving hemodialysis compared to that in normal subjects [15,16]. Although the urinary PF4 level has been examined in various forms of glomerulonephritis [17,18], the results have been inconclusive. Currently, little is known about the role of PF4 in pediatric CKD and its association with CVD risk.

PF4 is released into the circulating blood by activated platelets, and its degradation products are eliminated in the urine [11]. Recently, biomarkers in blood normalized to kidney function have appeared as new biomarkers and are considered to reflect the endogenous level more precisely than the blood level [19]. Urinary biomarkers are frequently reported as a normalized ratio to urinary creatinine. However, the origin of the urinary proteins is uncertain as they may potentially be blood-derived; alternatively, they may relate to increased renal expression or result from reduced tubular reabsorption. Accordingly, the diagnostic utility of the fractional excretion (FE) of analyzed biomarkers by normalizing both blood and urine biomarker levels against creatinine has been considered [20].

As such, the purpose of our study was to assess PF4 in the plasma and urine, along with its normalized ratio and FE, and determine their associations with ABPM and other CVD surrogate markers (i.e., LVMI, cIMT, and PWV) in children with CKD. Previously, we reported that nitric oxide (NO), a critical regulator of endothelial function, is involved in BP abnormalities in ABPM in CKD children [21]. As PF4 participates in the development of endothelial dysfunction [11,12,13], our secondary objective was to find a link between PF4 and NO in pediatric CKD.

## 2. Materials and Methods

### 2.1. Study Population

Children and adolescents aged 3 to 18 years diagnosed as having CKD according to the KDIGO 2012 clinical practice guideline [22] were recruited from our pediatric outpatient clinics between November 2018 and April 2022. Written informed consent was obtained from the participants and parents, and this study was approved by the Chang Gung Medical Foundation Institutional Review Boards (IRB) (201701735A3C501 and 202001973A3C601).

### 2.2. Assessment and Staging of CKD

We used the Schwartz formula for calculating the estimated glomerular filtration rate (eGFR) [23]. Children and adolescents were classified as CKD stages G1–G5 according to their eGFR. Participates were excluded for the following reasons: stage G5 CKD requiring dialysis or a kidney transplant, a history of congenital heart disease, pregnancy, or noncooperation. The etiologies of kidney diseases were allocated into two types, namely CAKUT and non-CAKUT [23]. The structural anomalies of CAKUT cover a duplicated collecting system, ureteropelvic junction obstruction, horseshoe kidney, renal agenesis, posterior urethral valves, kidney hypo-/dysplasia, multicystic kidney dysplasia, and ureter abnormalities [24].

### 2.3. Assay of Plasma and Urinary PF4

Peripheral venous blood samples were obtained after a 12 h fast. Spot urine samples were collected and kept at −80 °C until examination. Plasma and urinary CF4 levels were examined using an ELISA kit (catalog number: RAB0402, Merck, Darmstadt, Germany). The inter-assay and intra-assay coefficients of variations were <12% and <10%, respectively. Urinary PF4 was represented as a normalized ratio to urinary creatinine. Also, the kidney function-normalized plasma PF4/Cr ratio was calculated to reflect the endogenous PF4 concentration [19]. Furthermore, the fractional excretion (FE) of PF4 was calculated by normalizing both the plasma and urine PF4 levels against creatinine.

### 2.4. Office BP and ABPM Measurements

Office BP was obtained using a fully automated oscillometric sphygmomanometer after the children had rested for five minutes. Hypertension was diagnosed based on the 2017 AAP Guidelines [25]. Participants aged 6–18 years received 24 h ABPM using the Oscar II oscillometric monitor (SunTech Medical, Morrisville, NC, USA) [10]. An abnormal ABPM profile was determined, as follows, by sex and height: (1) daytime, nighttime, systolic BP (SBP), or diastolic BP (DBP) at the ≥95th percentile [26]; (2) a SBP or DBP load exceeding 20%; and (3) nocturnal reduction in the BP load of <10%.

### 2.5. Cardiovascular Assessments

Echocardiography and carotid ultrasound were performed at the same visit. All echocardiographic measurements were carried out using a Philips IE33 system device (Philips, Bothell, WA, USA). The left ventricular mass index (LVMI) was calculated by dividing the LV mass by height^2.7^ [27]. The carotid ultrasound assessment was performed using a ProSound α7 ultrasound device (Aloka Co., Tokyo, Japan), followed by analysis with an e-TRACKING system. The cIMT was determined using the edge-to-leading-edge technique for measuring the blood–intima and media–adventitia interfaces of the far wall.

### 2.6. Measurement of the NO Pathway

An ancillary study was conducted on a subgroup of 102 subjects who had undergone concurrent laboratory tests to determine PF4-related biomarkers and elements of the NO pathway. We determined the substrate (L-arginine) and endogenous inhibitors of NO synthase (NOS). Asymmetric and symmetric dimethylarginine (ADMA and SDMA) are known NOS inhibitors. To determine NO bioavailability, we calculated the L-arginine-to-ADMA ratio [28]. These elements of the NO pathway were analyzed via the HPLC method using homoarginine as an internal standard [21].

### 2.7. Statistical Analysis

Qualitative variables are expressed as a number (%) and quantitative variables as median and interquartile ranges (IQRs; 25th−75th percentile). Differences between groups were sought using the chi-square test for qualitative variables and the Mann–Whitney U-test for quantitative data. Numerical variables were adjusted for confounders via a multivariate analysis of variance. We used the logistic regression to study the associations of PF4-related biomarkers with the ABPM profile. The dependent variable (ABPM profile) is a categorical variable (normal or abnormal). The independent variable can be a categorical variable (i.e., sex and CAKUT) or a continuous variable (i.e., age and creatinine). Statistical significance was defined as a two-tailed *p* value of less than 0.05. SPSS version 16.0 (Chicago, IL, USA) was utilized for statistical analysis.

## 3. Results

### 3.1. Study Population

Table 1 lists the clinical and laboratory characteristics of the children with CKD in the study. In total, 139 children and adolescents with CKD stages G1–G4 were studied. The median age was 9.6 years, 56.8% were male, and 54.7% had CAKUT. There were 104 patients (74.8%) with CKD stage G1, 23 (16.5%) with CKD stage G2, 10 (7.2%) with CKD stage G3, and 2 (1.4%) with CKD stage G4. Most children and adolescents recruited in the present study are in the early stages of CKD. Compared with CAKUT, CKD non-CAKUT children were significantly older and had a greater body height, body weight, and body mass index. In addition, there was a higher eGFR, plasma concentrations of LDL and triglyceride, heavier proteinuria, and lower calcium and phosphate concentrations in children with non-CAKUT than in those with CAKUT. The diagnosis of hypertension accounting for 20.1% of CKD children was made via office BP readings, irrespective of CKD etiologies.

### 3.2. PF4-Related Biomarkers

**Figure 1:**
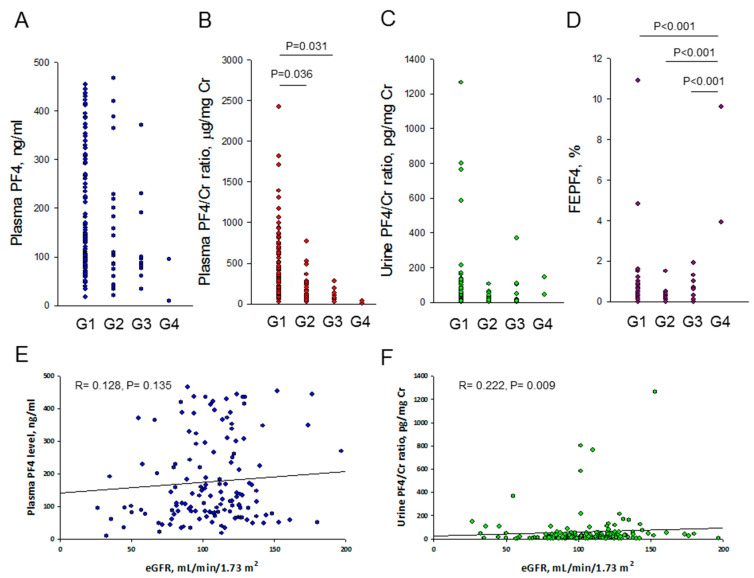
Comparison of (**A**) plasma PF4, (**B**) plasma PF4/Cr ratio, (**C**) urine PF4/Cr ratio, and (**D**) fractional excretion of PF4 (FEPF4) between those in the CKD stage G1–G4 group. Correlations between (**E**) plasma PF4 and eGFR and between the (**F**) urine PF4/Cr ratio and eGFR, as determined using Spearman’s correlation coefficient.

Figure 1 illustrates the plasma and urine levels of PF4. The plasma levels of PF4 were 137.4 (82.3–298.3), 108.1 (59.5–218.2), 92 (72.4–200.5), and 51.9 (8.9–94.9) ng/mL in subjects with CKD stage G1, G2, G3, and G4, respectively. The plasma levels of PF4 were comparable within stages G1–G4 (Figure 1A). After normalization to kidney function, the plasma PF4/Cr ratios were 298.8 (168.2–616.6), 155.7 (75.4–281), 89.7 (62.5–150.3), and 21.2 (4.6–37.8) μg/mg Cr in children with CKD stages G1, G2, G3, and G4, respectively. The plasma PF4/Cr ratio was more likely to be higher in children with CKD stage G1 compared to those with stage G2 (Figure 1B, *p* = 0.036) and G3 (Figure 1C, *p* = 0.031).

Figure 1C shows that the urine PF4/Cr ratio was not different among those with CKD stage G1-G4. However, among CKD children, the fractional excretion of PF4 was significantly higher in those who had stage 4 CKD than in those in other stages (Figure 1D, all *p* < 0.001). We next estimated the association of kidney function with PF4. A correlation between plasma PF4 and eGFR did not exist (*r =* 0.128; *p* = 0.135), while the correlation between the urine PF4/Cr ratio and eGFR became more significant in children with CKD (*r =* 0.222; *p* = 0.009).

Next, PF4-related biomarkers were compared by etiologies of CKD. Among children with non-CAKUT, the plasma PF4 in Table 2 also illustrates that there is no difference in the urine PF4/Cr ratio and FEPF4 between CAKUT and non-CAKUT children. However, the plasma PF4 level and PF4/Cr ratio were higher in children with non-CAKUT than those with CAKUT.

### 3.3. Cardiovascular Risk Assessment

Table 3 shows a subgroup of 102 CKD children who had received a comprehensive CV assessment. Although the LV mass was smaller in CKD children with CAKUT, the LVMI did not differ between CAKUT and non-CAKUT children. Regarding cIMT, augmentation index, and PWV, there was no difference between CAKUT and non-CAKUT children.

Table 4 illustrates the correlations between PF4-related biomarkers and CV markers in CKD children with CAKUT. We observed that the plasma PF4 (*r =* −0.498, *p* < 0.001) and plasma PF4/Cr ratio (*r =* −0.413, *p* = 0.002) showed significantly negative correlations with cIMT. In the correlation analysis, the plasma PF4 showed a significantly negative correlation with cIMT (*r =* −0.498, *p* < 0.001). The correlations between the PF4-related biomarkers with CV markers in CKD children without CAKUT were investigated, as shown in Table 5. The correlation analysis showed that the plasma PF4 was negatively correlated with the left ventricular mass (*r =* −0.321; *p* = 0.026), LVMI (*r =* −0.286, *p* = 0.049), cIMT (*r =* −0.498; *p* < 0.001), and PWV (*r =* −0.351; *p* = 0.013). In the non-CAKUT group, the plasma PF4/Cr ratio was negatively correlated with the LV mass (*r =* −0.511, *p* < 0.001) and PWV (*r =* −0.324; *p* = 0.023). In applications of regression analysis for the prediction model (*r =* 0.39; *p* = 0.045), plasma PF4 was still associated with cIMT (beta = −0.72, *p* = 0.007), controlling for age, sex, creatinine, and CAKUT.

### 3.4. PF4-Related Biomarkers vs. ABPM Profile

Table 6 shows the ABPM profile in children with CKD. Among 102 CKD patients who received an ABPM study, 70.6% of them (72/102) had at least one abnormality on ABPM, including 18 children (17.6%) with a 24 h BP ≥95th percentile, 11 children (10.8%) with a daytime BP ≥95th percentile, 22 children (21.6%) with a nighttime BP ≥95th percentile, 53 children (52%) with a BP load ≥25th percentile, and 53 children (52%) with a nocturnal reduction in the BP load of <10%. With respect to the association between the PF4-related parameters and ABPM profile, we observed that the plasma PF4 concentration was significantly lower in children with CKD accompanied by an abnormal 24 h BP and nocturnal non-dipping status. After normalization to kidney function, the plasma PF4/Cr ratio was lower in CKD children with a high 24 h BP, high daytime BP, high BP load, and nocturnal non-dipping status (Table 6). The urine PF4/Cr ratio and FEPF4 were not different between CKD children in the absence or presence of an abnormal ABPM profile.

We estimated the association of PF4-related biomarkers with the ABPM profile (Table 7). After multivariable adjustments for the confounders, such as age, sex, creatinine, and CAKUT, the plasma PF4 (aOR, 0.98; 95% CI, 0.96–0.99; *p* = 0.009) and plasma PF4/Cr ratio (aOR, 1.014; 95% CI, 1.004–1.024; *p* = 0.008) were independent factors for abnormal ABPM. In addition, the plasma PF4 (aOR, 0.99; 95% CI, 0.98–0.99; *p* = 0.036) and plasma PF4/Cr ratio (aOR, 1.005; 95% CI, 1–1.01; *p* = 0.049) were associated with an abnormal BP load in the adjusted regression model. Moreover, the plasma PF4/Cr ratio was an independent risk factor for night dipping after adjustments for the confounders (aOR, 1.007; 95% CI, 1–1.014; *p* = 0.038).

We next analyzed the correlations between PF4-related biomarkers and the NO pathway (Table 8). The ADMA was positive correlated with the plasma PF4 concentration (*r =* 0.241, *p* = 0.015) and PF4/Cr ratio (*r =* 0.262, *p* = 0.008) but negatively correlated with FEPF4 (*r =* −0.267, *p* = 0.007). Conversely, the AAR was negatively correlated with the plasma PF4 concentration (*r =* −0.377, *p* < 0.001) and the PF4/Cr ratio (*r =* −0.425, *p* < 0.001) but positively correlated with FEPF4 (*r =* 0.384, *p* < 0.001). No correlations exist between PF4-related biomarkers and L-arginine as well as SDMA. Our findings propose a link between PF4-related biomarkers and the NO pathway.

## 4. Discussion

The current study is among the earliest to investigate potential protein biomarkers in pediatric CKD using a proteomic approach [6,29]. To the best of our knowledge, our study is the first to assess the link between PF4 and the subclinical CVD risk in pediatric CKD. In agreement with our former study [10], we identified PF4-related biomarkers and found that they may have a differential impact on CKD children with or without CAKUT. These PF4-related biomarkers were able to correlate with the severity of CKD, relate to CVD structural and functional markers, and differentiate an abnormal ABPM profile from a normal one. These observations suggest that the pathogenic mechanisms underlying CVD in pediatric CKD may be attributed to PF4.

We observed that the plasma PF4 level is comparable to that reported in children without an age-dependent variation [30]. Whether or not the plasma PF4 level is influenced by kidney function remains inconclusive [15,16]. In our analyses of PF4-related biomarkers in pediatric CKD, we found that the plasma PF4 level was not different among the four stages of CKD and did not correlate with the eGFR. Considering the potential contribution of kidney to plasma PF4, a function-normalized PF4/Cr ratio might be better representative of endogenous PF4 and correlate with CKD comorbidities. We found that the plasma PF4/Cr ratio was more likely to be higher in children with normal kidney function compared to those with decreased kidney function. As PF4 is mainly degraded rather than eliminated in the urine [11], our data suggest that the breakdown of PF4 may depend on the severity of CKD in these children.

Moreover, FEPF4 was calculated to provide a precise picture of the renal handling of a filtered molecule in this study. Fractional excretion is chiefly influenced by two major determinants, renal production and tubular reabsorption. Although the FEPF4 was significantly elevated in the CKD stage 4 group, it is a bit difficult to draw conclusions based on such small case numbers. Whether or not worse CKD causes an overproduction of PF4 in the kidneys or a reduction in the tubular reabsorption of PF4 awaits further clarification.

The presence of abnormal surrogate markers of CVD including an increased LVMI, cIMT, and PWV has been linked to hypertension in pediatric CKD [31]. A correlation between plasma PF4 and these surrogate markers was associated with a greater burden of subclinical CVD in CKD children without CAKUT. However, in the CAKUT group, plasma PF4 only correlated with cIMT. These findings suggest that PF4 may work differentially in CAKUT and non-CAKUT, and we need to determine their mechanisms underlying CVD.

Many reports have demonstrated that ABPM is superior to office BP in relation to target organ damage and is better at detecting hypertension in pediatric CKD [32]. In agreement with prior work, we identified that approximately 70% of CKD children had BP abnormalities in ABPM, while only one fifth of them were diagnosed with hypertension according to office BP. In the current study, the plasma PF4/Cr ratio was decreased in CKD children with abnormal ABPM, including a high 24 h BP, high daytime BP, high BP load, and nocturnal non-dipping. After adjustments for the confounders, the plasma PF4/Cr ratio was still an independent risk factor for a high BP load and nocturnal non-dipping. Interestingly, a previous study revealed that the plasma PF4 level was higher in hypertensive patients than in normotensive controls and increased with the stage of hypertension [33]. Although how PF4 influences BP remains unclear, our data suggest that NO might be a possible link between them.

NO is recognized as the most important endothelial-derived relaxing factor. Deficient NO causes a loss of endothelium-dependent vasorelaxation, leading to endothelial dysfunction. In pediatric CKD, high BP and CVD risk are related to low NO bioavailability [21]. It is known that low AAR and high ADMA represent decreased NO bioactivity in favor of hypertension. In the current study, both the PF4 level and PF4/Cr ratio in the plasma are positively correlated with ADMA and negatively correlated with the L-arginine-to-ADMA ratio. In view of prior research showing that NO can increase PF4 levels [34], PF4 may provoke NO-mediated compensatory responses in CKD children following the elevation in BP. A previous study showed that circulating PF4 can be transported into vessels and present in atheromatous plaque [14]. Clearly, extended work is needed to explore how PF4 interacts with NO to modulate cellular events underlying CVD pathogenesis in children with CKD.

PF4 has multiple roles in diseases. PF4 probably also contributes to oxidative stress. As previously shown, PF4 can induce a monocyte oxidative burst, which participates in the pathogenesis of oxidative-stress-mediated vascular damage [35]. In addition, as a platelet-derived chemokine, PF4 can promote local inflammatory processes at sites of vascular injury [36]. Considering oxidative stress and inflammation are involved in the development of CVD in patients with CKD [37], there is a growing attempt to elucidate whether or not the interplay between PF4 and these mechanisms may play a role in CVD in pediatric CKD.

Several limitations should be considered in this study. First, we mainly draw a conclusion based on the present cross-sectional study; hence, it is hard to establish a cause-and-effect relationship between PF4 and CVD in CKD. Further research is needed to establish causal relationships for the existing correlations. Second, the small sample size recruited from a single medical center might have limited generalizability to the overall pediatric population with CKD. Third, we did not recruit healthy children in this study, as children with normal kidney function in CKD stage G1 served as the control for comparison. Whether or not PP4 levels differ between children with and without CKD deserves to be illuminated. Lastly, there is a lack of comparison of the clinical value of PF4 and that of other traditional CVD biomarkers. Further studies are required to confirm and validate PF4 as a potential biomarker using large sample sizes and should be reproducible, specific, and sensitive.

## 5. Conclusions

In conclusion, plasma PF4 is negatively linked with cIMT and ABPM abnormalities, revealing that PF4 may serve as a biomarker for subclinical CVD in pediatric CKD. PF4 in plasma showed markedly better discriminative performance for ABPM abnormalities and is better linked to CVD surrogate markers than its urine biomarker. Longitudinal studies are required to estimate the performance of these PF4-related biomarkers in predicting CVD events and CKD progression.

## Figures and Tables

**Table 1:** Clinical and laboratory characteristics of the study population.

Characteristics	Overall	CAKUT	Non-CAKUT	
	N = 139	N = 76	N = 63	*p* Value
Age, years	9.6 (5.6–14.2)	9 (5–13.1)	11 (6.5–15.4)	0.03
Male sex	79 (56.8%)	46 (60.5%)	33 (52.4%)	0.391
CKD stage				0.429
Stage G1	104 (74.8%)	53 (69.7%)	51 (81%)	
Stage G2	23 (16.5%)	16 (21.1%)	7 (11.1%)	
Stage G3	10 (7.2%)	6 (7.9%)	4 (6.3%)	
Stage G4	2 (1.4%)	1 (1.3%)	1 (1.6%)	
Body height, cm	131.8 (114.4–159.9)	129.6 (108.2–153.5)	143.7 (120–164)	0.032
Body weight, kg	32.2 (20–51)	24.9 (18.9–45.8)	36.4 (22.3–54.7)	0.029
Body mass index (kg/m^2^)	17.4 (15.6–20.4)	16.8 (14.8–19.1)	18 (16.3–21.2)	0.032
Systolic blood pressure (mmHg)	109 (100–120)	109 (100–118)	110 (100–122)	0.351
Diastolic blood pressure (mmHg)	68 (63–75)	67 (60–74)	69 (64–78)	0.156
Hypertension (by office BP)	28 (20.1%)	13 (17.1%)	15 (23.8%)	0.521
Blood urea nitrogen (mg/dL)	12 (10–14)	13 (10.3–15)	11 (10–13)	0.054
Creatinine (mg/dL)	0.5 (0.42–0.69)	0.51 (0.43–0.72)	0.5 (0.4–0.61)	0.313
eGFR (mL/min/1.73 m^2^)	108 (89–125)	102 (85–116)	120 (95–132)	<0.001
Fasting glucose (mg/dL)	87 (83–92)	87.4 (84–91.8)	87 (82.5–92)	0.515
Total cholesterol (mg/dL)	164 (146–187)	162 (143–179)	172 (148–198)	0.091
Low-density lipoprotein (mg/dL)	88 (71–107)	83 (68–98)	97 (80–123)	0.001
Triglyceride (mg/dL)	67 (49–102)	61 (47–78)	82 (61–133)	0.001
Uric acid (mg/dL)	5.3 (4–6.3)	5.1 (4–6.2)	5.7 (4.3–6.9)	0.117
Sodium (mEq/L)	141 (137–142)	141 (140–142)	140 (139–142)	0.069
Potassium (mEq/L)	4.4 (4.2–4.5)	4.4 (4.2–4.6)	4.3 (4.1–4.5)	0.186
Calcium (mg/dL)	9.7 (9.4–9.9)	9.8 (9.5–10)	9.5 (9.1–9.8)	0.001
Phosphate (mg/dL)	4.9 (4.5–5.3)	5.1 (4.8–5.4)	4.7 (4.3–5.2)	0.007
UPCR (mg/g)	63.2 (42.5–261.6)	52 (37.9–72)	200.8 (54.2–944.7)	<0.001
Hemoglobin (g/dL)	13.4 (12.5–14.3)	13.5 (12.5–14.3)	13.2 (12.4–14.5)	0.628
Platelet (10^9^/L)	287 (253–331)	292 (259–326)	283 (242–346)	0.881

Data presented as N (%) or median (IQR). CAKUT = congenital anomalies of the kidneys and urinary tract; eGFR = estimated glomerular filtration rate; UPCR = urine protein-to-creatinine ratio.

**Table 2:** PF4 concentration in the plasma and urine, Cr normalized ratio, and fractional excretion of PF4 in children with CKD.

PF4	CAKUT	Non-CAKUT
	N = 76	N = 63
Plasma PF4 level (ng/mL)	102.2 (70.8–197.2)	165.8 (86.1–337.6) *
Plasma PF4/Cr ratio (μg/mg Cr)	21.9 (10.8–39.7)	27.9 (16.4–63.9) *
Urine PF4/Cr ratio (pg/mg Cr)	190 (80–470)	240 (90–530)
FEPF4 (%)	0.95 (0.4–2.33)	0.5 (0.2–2.5)

Data presented as N (%) or median (IQR). CAKUT = congenital anomalies of the kidneys and urinary tract. * *p* < 0.05, determined via the Mann–Whitney U-test.

**Table 3:** Cardiovascular markers in children with CKD.

CV Markers	CAKUT	Non-CAKUT
	*n* = 53	*n* = 49
Left ventricular mass (g)	78.9 (57.4–106)	92.5 (65.4–127.8) *
LVMI (g/m^2.7^)	30.9 (26.1–37.9)	32.2 (27–38.2)
cIMT	0.34 (0.3–0.44)	0.33 (0.31–0.42)
Augmentation index	−3.7 (−7.9–−0.6)	−5.5 (−11.5–−1.05)
PWV	3.7 (3.3–4.1)	3.8 (3.5–4.4)

Data are medians (IQR). LVMI = left ventricular mass index. cIMT = carotid artery intima–media thickness. PWV = pulse wave velocity. * *p* < 0.05 in the Mann–Whitney U-test.

**Table 4:** Correlation between PF4-related biomarkers and cardiovascular markers in CKD children with CAKUT.

Cardiovascular Markers	Plasma PF4		Plasma PF4/Cr Ratio		Urine PF4/Cr Ratio		FEPF4	
	*r*	*p*	*r*	*p*	*r*	*p*	*r*	*p*
Left ventricular mass	0	0.999	−0.234	0.092	−0.085	0.545	0.003	0.985
LVMI	−0.098	0.486	−0.037	0.793	−0.089	0.527	−0.026	0.855
cIMT	−0.498	<0.001 *	−0.413	0.002 *	−0.084	0.552	0.23	0.097
Augmentation index	−0.113	0.245	0.11	0.434	0.105	0.453	0.041	0.772
PWV	0.163	0.245	−0.104	0.458	−0.017	0.906	−0.019	0.895

N = 53; * *p* < 0.05, determined via Spearman’s correlation coefficient.

**Table 5:** Correlation between PF4-related biomarkers and cardiovascular markers in CKD children with non-CAKUT.

Cardiovascular Markers	Plasma PF4		Plasma PF4/Cr Ratio		Urine PF4/Cr Ratio		FEPF4	
	*r*	*p*	*r*	*p*	*r*	*p*	*r*	*p*
Left ventricular mass	−0.321	0.026 *	−0.511	<0.001 *	0.021	0.886	0.226	0.112
LVMI	−0.286	0.049 *	−0.255	0.08	0.003	0.985	0.155	0.293
cIMT	−0.335	0.019 *	−0.254	0.078	0.005	0.971	0.196	0.177
Augmentation index	0.223	0.124	0.261	0.07	0.055	0.705	−0.139	0.34
PWV	−0.351	0.013 *	−0.324	0.023 *	−0.11	0.452	0.035	0.81

N = 49; * *p* < 0.05, determined via Spearman’s correlation coefficient.

**Table 6:** PF4-related biomarkers vs. ABPM profile in children and adolescents with CKD.

ABPM Profile	*n*	Plasma PF4	Plasma PF4/Cr Ratio	Urine PF4/Cr Ratio	FEPF4
24 h BP					
Abnormal	18	83.7 (48.8–183) *	113.3 (60.1–221.8) *	9 (4–82.3)	0.1 (0–1.5)
Normal	84	142.3 (81.5–307.3)	264.8 (137.7–514.6)	17.5 (8–39)	0.1 (0–0.2)
Daytime BP					
Abnormal	11	85.1 (59.5–109.9)	115.7 (64.7–194.4) *	8 (4–52)	0.1 (0–3.9)
Normal	91	140.7 (76.2–309.9)	261.7 (129.1–516.4)	17 (8–41)	0.1 (0–0.2)
Nightime BP					
Abnormal	22	90 (56–244.9)	140.2 (65–433.7)	18.5 (4.8–80)	0.1 (0–1.1)
Normal	80	138.8 (81.5–292.1)	254.5 (134.8–503.2)	15.5 (7.3–38.8)	0.1 (0–0.2)
BP load					
Abnormal	53	94.3 (60–270.4)	165.9 (74.8–438.9) *	14 (7–50)	0.1 (0–0.3)
Normal	49	154.4 (98.2–284.4)	278.6 (174.8–516.8)	19(8–38.5)	0.1(0–0.2)
Night dipping					
Abnormal	53	181.6 (84.3–338.1) *	310.5 (126–647.2) *	17 (7.5–51.5)	0.1 (0–0.25)
Normal	49	100.8 (65.5–166.5)	210.1 (106.5–312.5)	16 (7–40)	0.1 (0–0.2)
ABPM profile					
Abnormal	72	112.2 (75.3–285.8)	216.1 (104.6–485.5)	14 (7–46.3)	0.1 (0–0.2)
Normal	30	135 (67.3–256.6)	264.8 (151.8–437)	20 (8.8–41.3)	0.1 (0–0.23)

Data given as medians (IQR) or n (%). N = 102. * *p* < 0.05 abnormal vs. normal group, determined via the Mann–Whitney U-test.

**Table 7:** Logistic regression model estimates of the association between PF4-related biomarkers and surrogate markers of CV risk.

	aOR *	95% CI	*p* Value
ABPM profile			
Plasma PF4	0.98	0.96–0.99	0.009
Plasma PF4/Cr ratio	1.014	1.004–1.024	0.008
BP load			
Plasma PF4	0.99	0.98–0.99	0.036
Plasma PF4/Cr ratio	1.005	1–1.01	0.049
Night dipping			
Plasma PF4/Cr ratio	1.007	1–1.014	0.038

* Adjusted for age, sex, creatinine, and CAKUT.

**Table 8:** Correlations between PF4-related biomarkers and the NO pathway.

Cardiovascular Markers	Plasma PF4		Plasma PF4/Cr Ratio		Urine PF4/Cr Ratio		FEPF4	
	*r*	*p*	*r*	*p*	*r*	*p*	*r*	*p*
L-arginine	−0.093	0.351	−0.161	0.106	0.015	0.884	0.115	0.25
ADMA	0.241	0.015 *	0.262	0.008 *	−0.154	0.122	−0.267	0.007 *
SDMA	0.172	0.085	0.077	0.442	0.01	0.918	−0.012	0.903
L-arginine-to-ADMA ratio	−0.377	<0.001 *	−0.425	<0.001 *	0.179	0.072	0.384	<0.001 *

N = 102; * *p* < 0.05, determined via Spearman’s correlation coefficient.

## Data Availability

Data are contained within the article.
